# Changes in Calprotectin (S100A8-A9) and Aldolase in the Saliva of Horses with Equine Gastric Ulcer Syndrome

**DOI:** 10.3390/ani13081367

**Published:** 2023-04-16

**Authors:** Alberto Muñoz-Prieto, María Dolores Contreras-Aguilar, José Joaquín Cerón, Ignacio Ayala de la Peña, María Martín-Cuervo, Peter David Eckersall, Ida-Marie Holm Henriksen, Fernando Tecles, Sanni Hansen

**Affiliations:** 1 Interdisciplinary Laboratory of Clinical Analysis (INTERLAB-UMU), Department of Animal Medicine and Surgery, Veterinary School, Regional Campus of International Excellence Mare Nostrum, University of Murcia, 30100 Murcia, Spain; 2 Animal Medicine, Faculty of Veterinary Medicine of Cáceres, University of Extremadura, Av. de la Universidad S-N, 10005 Cáceres, Spain; 3 School of Biodiversity, One Health and Veterinary Medicine, University of Glasgow, Bearsden Rd, Glasgow G61 1QH, UK; 4 Department of Veterinary Clinical Sciences, Veterinary School of Medicine, Sektion Medicine and Surgery, University of Copenhagen, Hoejbakkegaard Allé 5, DK-2630 Høje-Taastrup, Denmark

**Keywords:** saliva, EGUS, ESGD, EGGD, calprotectin, aldolase, biomarkers

## Abstract

**Simple Summary:**

Equine gastric ulcer syndrome (EGUS) is a frequent disease that considerably reduces the quality of life of horses, while also affecting their physical performance, even when the disease is subclinical. In this study, we hypothesized that two analytes, calprotectin (CALP) and aldolase, could be measured in saliva with commercially available assays and be potential biomarkers of EGUS in horses. We investigated the changes in the salivary CALP and aldolase in 131 horses divided into 5 different groups: controls (healthy horses), with equine squamous gastric disease (ESGD), with equine glandular gastric disease (EGGD), with combined ESGD and EGGD, and horses with other intestinal pathologies but clinical signs similar to EGUS. The two assays were precise and accurate, and, in both cases, they showed differences between horses with EGUS and healthy horses, but they did not show significant differences between horses with EGUS and horses with other diseases. Our data showed that CALP has a high ability to differentiate between healthy horses and horses with EGUS. Therefore, it could have potential use as a biomarker since a value of CALP in the range of healthy individuals could indicate that the animal is not likely to have EGUS at gastroscopy.

**Abstract:**

Equine gastric ulcer syndrome (EGUS) is a highly prevalent disease that affects horses worldwide. Within EGUS, two different forms have been described: equine squamous gastric disease (ESGD) and equine glandular gastric disease (EGGD). The associated clinical signs cause detrimental activity performance, reducing the quality of life of animals. Saliva can contain biomarkers for EGUS that could be potentially used as a complementary tool for diagnosis. The objective of this work was to evaluate the measurements of calprotectin (CALP) and aldolase in the saliva of horses as potential biomarkers of EGUS. For this purpose, automated assays for the quantification of these two proteins were analytically validated and applied for detecting EGUS in a total of 131 horses divided into 5 groups: healthy horses, ESGD, EGGD, combined ESGD and EGGD, and horses with other intestinal pathologies. The assays showed good precision and accuracy in analytical validation, and they were able to discriminate between horses with EGUS and healthy horses, especially in the case of CALP, although they did not show significant differences between horses with EGUS and horses with other diseases. In conclusion, salivary CALP and aldolase can be determined in the saliva of horses and further studies are warranted to elucidate the potential of these analytes as biomarkers in EGUS.

## 1. Introduction

Equine gastric ulcer syndrome (EGUS) is a highly prevalent disease in horses that comprises two different entities: equine squamous gastric disease (ESGD) and equine glandular gastric disease (EGGD) [1]. Each of these two diseases has its own risk factors, pathogenesis, and treatment [2]. While ESGD is considered to result mainly from increased acid exposure, EGGD is thought to be related to the compromise of mucosal defence mechanisms [3]. The clinical signs most commonly associated with EGUS are poor appetite, weight loss, poor body condition, colic, poor performance and discomfort when tightening the girth [4]. However, horses can be affected by EGUS with no obvious clinical signs, or the signs can be subtle ones not recognized by the owners. Therefore, they could be considered healthy by their owners and trainers even though they have lesions only evidenced by gastroscopy [5]. 

A previous work found that EGUS produces changes in saliva composition [6]. When a panel of saliva analytes was studied in horses with EGUS, including different enzymes, metabolites, proteins, redox biomarkers, and minerals, it was found that some analytes such as adenosine deaminase (ADA), uric acid, triglycerides and calcium have the potential to detect EGUS in horses [6]. In addition, analytes related to oxidative stress showed the ability to differentiate between horses with EGGD and healthy horses [6,7]. These results indicate the potential of saliva to reveal the changes occurring in this disease with the advantage of not producing stress in horses [8]. 

In a previous study, in which saliva proteins were evaluated using proteomic analysis, calprotectin (S100A8-A9, CALP) was found to be increased in horses with EGUS when compared to healthy horses. In addition, another protein, aldolase, showed significant differences between horses diagnosed with EGGD and ESGD [9]. CALP is a calcium-binding S100 leucocyte protein related to innate immune response and inflammation [10,11,12]. Aldolase belongs to a family of proteins involved in gluconeogenesis and glycolysis [13]. These two proteins have been related to gastrointestinal diseases in other species. For example, faecal CALP is a marker of intestinal inflammation in humans [11,12] and aldolase increases in the serum of patients with colorectal cancer [14]. In equine patients, increases in CALP have been detected in the tissues affected by large colon ischemia and laminitis [15,16].

Currently, there are commercially available assays that allow the measurement of CALP [17] and aldolase [18], having the advantages associated with automation, such as higher precision and faster sample processing. The hypotheses of this work are that CALP and aldolase can be measured in the saliva of horses with commercially available assays and, furthermore, that these assays can detect the changes in these analytes that occur in horses with EGUS. Therefore, the objectives of this study were firstly to validate, from an analytical point of view, two commercially available assays for the measurement of CALP and aldolase in the saliva of horses, and secondly, to study the possible changes in these analytes in horses with both types of EGUS (ESGD and EGGD) compared to healthy horses. Additionally, the ability of these analytes to differentiate EGUS from non-EGUS horses but with compatible clinical signs due to other diseases was investigated.

## 2. Materials and Methods

### 2.1. Animals

Banked samples of saliva from a previous study [6] and other additional samples, all collected during 2022, were used for the measurements of CALP and aldolase in the following groups:

- EGUS groups: animals with compatible signs according to the criteria established previously [6] were further divided into the different types of EGUS:
  ○ ESGD group: horses with a score ≥ 1 on the 4-point ESGD gradation scale at gastroscopy according to the European College of Equine Internal Medicine (ECEIM) Consensus Statement [3].
  ○ EGGD group: since a validated grading system for EGGD is not widely accepted, the description of the anatomical lesions compatible with the disease determined by gastroscopy was used for diagnosis, as recommended by ECEIM [3].
  ○ Combination of ESGD and EGGD: horses that presented a positive diagnosis for ESGD and EGGD at the same time.
- Non-EGUS group: In the case of animals with compatible signs of EGUS but without compatible gastroscopic images. In this group, further diagnostic exams were assessed in those cases required. These exams were per rectum exploration, transabdominal ultrasonography, intestinal biopsies, abdominocentesis or exploratory laparotomy.
- Healthy control group: Only animals with no signs of any pathology were included in this group. These horses showed no alteration during the physical examination and haematological, biochemical, and gastroscopic analysis.

The body condition score of the animals of all groups was catalogued based on a previously described 9-point scale [19].

### 2.2. Saliva Sampling

Saliva was obtained from all horses before performing intravenous sedation and gastroscopy when the horses were placed in the examination stock using a sponge, as previously described [6]. A period of fasting of 12 h was established before taking the saliva sample. Samples were kept frozen at −80 °C until analysed. The degree of dirtiness accepted for saliva was 0 or 1 according to the previously reported colour scale (0–4 score) [20].

### 2.3. Measurements of Calprotectin Concentration and Aldolase Activity

Saliva CALP was determined with the BÜHLMANN fCal Turbo^®^ assay (BÜHLMANN, Laboratories AG, Schönenbuch, Switzerland). Initial calibration of the assay was performed with a control material of human calprotectin (REF 1219, Gentian SA, Moss, Norway) (Condition A), and then a secondary calibrator consisting of saliva samples measured with Condition A was employed to perform a complete analytical validation and the subsequent sample analysis. Aldolase activity was measured using a commercially available reagent kit based on an enzymatic reaction using fructose 1–6 diphosphate as substrate (Aldolase, Randox Laboratories Ltd., Crumlin, UK). These assays were applied to a Beckman Coulter AU 400 autoanalyzer (Olympus Diagnostica GmbH AU 400, Beckman Coulter, Ennis, Ireland) following the manufacturer’s recommendations.

The CALP and aldolase automated assays were validated before their use in equine saliva using the aliquots of the banked samples indicated above by the study of:

- Precision: The intra- and inter-assay coefficient of variation (CV) were obtained from two saliva samples with high and low values, respectively.
- Accuracy: This was assessed through the study of linearity after serial dilutions of a saliva sample with a high level of the analyte diluted with ultrapure water. In addition, recovery studies in which purified CALP or aldolase using the control material of the assays were spiked with a saliva sample to reach three different analyte concentrations were performed.
- Limit of quantification (LoQ): based on the lowest CALP concentration and aldolase activity that had an imprecision lower than 20%.
- Limit of detection (LD): This was determined by the lowest concentration of CALP and activity of aldolase that could be distinguished from a specimen of zero value, calculated as a mean value plus 2 standard deviations of 12 replicate determinations of the zero standards (ultrapure water).

### 2.4. Statistical Analysis

The assays’ coefficients of variation (CV) were calculated as the standard deviation (SD) divided by the mean value of analysed replicates × 100%. The lower limits of quantification and linearity under dilution were calculated using the Excel spreadsheet for MAC (Excel 2020, Microsoft Corporation, Redmond, Washington, DC, USA).

The data distribution was evaluated with the Shapiro–Wilk test showing a non-parametric distribution. The differences between groups were assessed through the Kruskal–Wallis test, followed by multiple pairwise comparisons. Receiver operating characteristic (ROC) analyses were performed for CALP and aldolase to evaluate their optimal value to differentiate the EGUS group vs. healthy horses; and the EGUS group vs. horses with other diseases [21]. Sensitivity, specificity, and positive and negative likelihood ratios (LR+ and LR-, respectively) were obtained from the ROC analyses. Correlations between ESGD score and CALP and aldolase in horses with ESGD and ESGD+EGGD were analysed through the Spearman test. A correlation coefficient ≥ 0.7 was considered a strong correlation in the comparison. Statistical analysis was performed with the commercial statistics packages SPSS (IBM SPSS Statistics for Windows, v28.0. IBM Corp. Armonk, NY, USA) and GraphPad (GraphPad Prism 9 for macOS). Values of *p* < 0.05 were selected to indicate significance for all analyses.

## 3. Results

### 3.1. Population Included

The population included 131 horses. The healthy group was composed of 15 animals (5 mares, 10 geldings) of different breeds with a median age of 8.6 (range: 4–18) years old and a BCS of 5.4 (range: 4–8). A total of 95 horses (45 mares, 50 geldings) of different breeds were included in the EGUS group, with a median age of 10.7 (range 4–24) years and a BCS of 5.6 (range 4–8). This group included 25 horses with ESGD, 32 with EGGD, and 38 with both ESGD + EGGD. A group of horses with other intestinal diseases different from EGUS was composed of 21 animals of diverse breeds (11 mares, 10 geldings) with a median of 9.5 (range 4–21) years old and a BCS of 5.6 (range 3–7). Information about the diagnoses in these animals is indicated in Table S1. There were no statistically significant differences when comparing age and BCS in the groups.

### 3.2. Assays Validation

**Figure 1:**
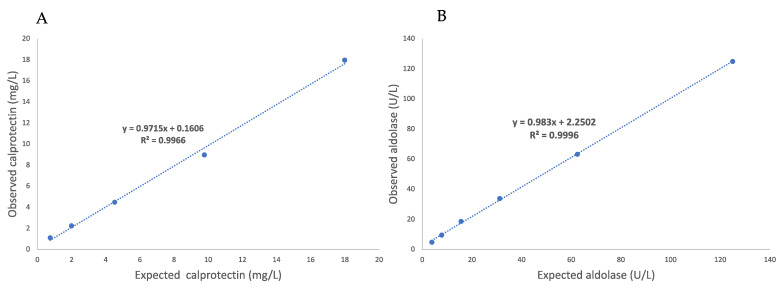
Linearity under dilution study of calprotectin (**A**) and aldolase (**B**) measurements in the saliva of horses.

**Figure 2:**
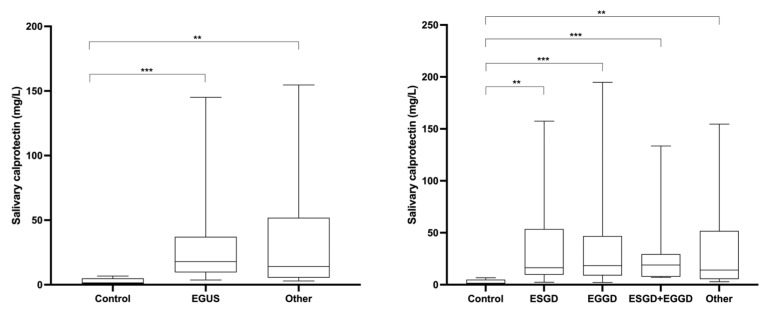
Salivary calprotectin concentrations observed in healthy horses (Control), horses diagnosed with equine gastric ulcer syndrome (EGUS), and horses with similar symptomatology of EGUS but diagnosed with other gastrointestinal disorders (Other). The median values are represented by the lines, the 10–90 percentiles by the boxes, and the range by the whiskers. **: *p* < 0.01; ***: *p* < 0.001.

The mean intra- and inter-assay CVs were 0.85 and 3.75 % for CALP and 5.27 and 6.89 % for aldolase (Table 1). The recovery rates ranged from 89.8% to 119.5% for CALP measurements, and from 97.1 to 100.9% in the case of aldolase (Table 2). Linear regression equations with a coefficient of determination close to 1 were observed after serial dilutions in both assays (Figure 1 and Figure 2). The LoQ was set at 0.01 mg/L for CALP and at 3.8 U/L for aldolase, and the LD of the assays was 1.37 U/L for aldolase and could not be calculated for CALP since all measurements gave a value of zero.

### 3.3. Changes in Calprotectin in the Saliva of Horses with EGUS

**Figure 3:**
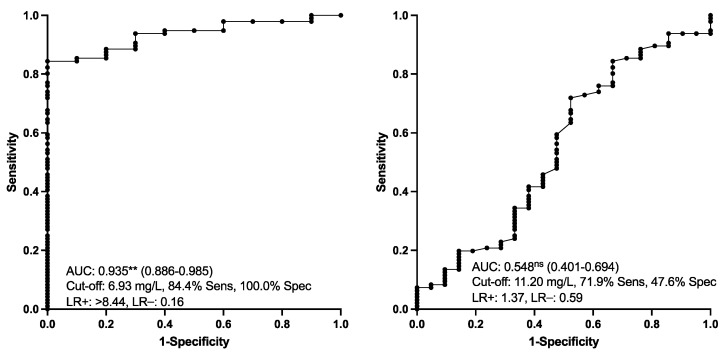
Salivary calprotectin concentrations. Receiver operating characteristic (ROC) analysis for discriminating between animals with EGUS from healthy ones (**left**) and EGUS from other diseases (**right**). AUC, the area under the curve (95% confidence interval within parenthesis); Sens, sensitivity; Spec, specificity; LR, likelihood ratio. Statistical analysis: **: *p* < 0.01; ns: *p* > 0.05).

Salivary CALP concentrations in the different groups of horses are indicated in Figure 2. CALP measured in horses with EGUS (group ESGD, EGGD and both EGGD and ESGD) was significantly higher than in healthy individuals. An increment in CALP concentrations was also observed in the saliva of horses with other pathologies different from EGUS but with compatible clinical signs. No significant differences were found in CALP between horses with EGGD and ESGD and between horses with EGUS and horses with other diseases different from EGUS. The results of the ROC analyses are shown in Figure 3. Salivary CALP was able to discriminate EGUS from healthy animals with >84% sensitivity and 100% specificity but was not able to differentiate EGUS from horses with other different disorders. 

A significant but weak correlation (Spearman correlation coefficient 0.316, *p* < 0.05) was observed between the salivary CALP concentration and ESGD grade.

### 3.4. Changes in Aldolase in the Saliva of Horses with EGUS

**Figure 4:**
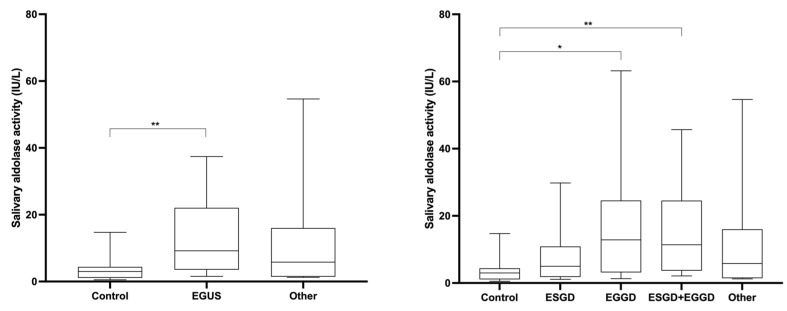
Salivary aldolase activity observed in healthy horses (Control), horses diagnosed with equine gastric ulcer syndrome (EGUS), and horses with similar symptomatology of EGUS but diagnosed with other gastrointestinal disorders (Other). The median values are represented by the lines, the 10–90 percentiles by the boxes, and the range by the whiskers. *: *p* < 0.05; **: *p* < 0.01.

**Figure 5:**
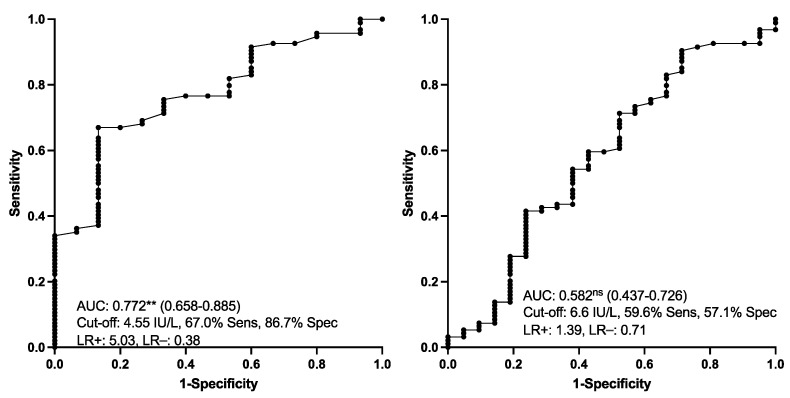
Salivary aldolase activity. Receiver operating characteristic (ROC) analysis for discriminating between animals with EGUS from healthy ones (**left**) and EGUS from other gastrointestinal diseases (**right**). AUC, the area under the curve (95% confidence interval within parenthesis); Sens, sensitivity; Spec, specificity; LR, likelihood ratio. Statistical analysis: **: *p* < 0.01; ns: *p* > 0.05).

Aldolase activity was significantly higher in horses with EGUS compared with healthy horses (Figure 4). No significant differences were found in aldolase between horses with EGGD and ESGD and between horses with EGUS and horses with other diseases different from EGUS. The results of the ROC analyses are shown in Figure 5. Salivary aldolase was able to discriminate EGUS from healthy animals with 67% sensitivity and 86.7% specificity, but no usefulness was found to differentiate EGUS from other diseases. 

No correlation (Spearman correlation coefficient 0.127, *p* > 0.05) was observed between salivary aldolase activity and ESGD grade.

## 4. Discussion

In this report, commercially available automated assays for the measurement of CALP and aldolase in the saliva of horses were validated. These assays were precise and accurate and were able to detect both analytes in the saliva of horses. The assay for the measurement of CALP was based on immunoturbidimetry and used a polyclonal antibody against human CALP. Equine and human CALP have a homology of 76% observed in the Basic Local Alignment Search Tool (BLAST) from NCBI [22] and this could explain why this assay also worked in horses. The assay for aldolase measurements is spectrophotometric and based on the enzymic activity of aldolase. Therefore, it is expected that it can be used in different species. Both assays can be automated, with the advantages associated with higher precision and sample throughput.

CALP (S100A8/A9) is a dimer complex of S100A8 and S100A9. The increase found in our study in horses with EGUS would agree with reports in humans where CALP in faeces increased in patients with erosive gastritis by around 3.8-fold and in patients with peptic ulcers by 6.9-fold compared with individuals with normal endoscopic findings [11]. The increase in this protein in EGGD could be related to the activation of the immune system that occurs in this disease [23]. In agreement with this finding, CALP was increased in a previous proteomic study in saliva in horses with EGGD, together with other proteins such as TMPRSS11D, joining (J) chain, and adenosyl homocysteinase that are involved in the regulation and activation of the immune system [24]. In a previous report, S100A9 was linked to inflammation since extracellular S100A9 may generate a chemotactic gradient that allows for neutrophil recruitment [25]. It is also interesting to point out that S100 calgranulin proteins (S100A8, S100A9 and S100A12) in children were virtually absent in normal gastric mucosa but were highly expressed when the mucosa was infected with *Helicobacter pylori* [26].

The increases in CALP found in horses with ESGD could be influenced by the hyperkeratosis of the squamous mucosal cells that appears in this disease, as significant increases in S-100 protein-positive dendritic cells of the gastric mucosa have been observed in situations of epithelial cell proliferation [27]. Some of the proteins found to be increased in ESGD in a previous proteomic study in saliva, such as serpin B5, WDR1, phosphoglycerate kinase 1 (PGK1), and keratins 15 and 4, have as a common feature the regulation of the growth of squamous epithelial cells [6]. In addition, CALP can activate the nicotinamide adenine dinucleotide phosphate (NADPH) oxidase, which is a membrane enzyme responsible for producing different reactive oxygen species (ROS) or free radical molecules to eliminate intracellular pathogens and microorganisms. Therefore, it could be postulated that the overexpression of CALP could lead to enhanced NADPH oxidase activity and reactive oxygen species (ROS) production and subsequent mucosal damage [28]. The results of our study agree with the previously reported increase in this protein in saliva detected by proteomics in horses with ESGD and EGGD compared with healthy horses (8).

ROC data showed that CALP has a high ability to differentiate between healthy horses and horses with EGUS. Therefore, as reported with other analytes such as ADA, CALP could have potential use as a biomarker since a value of CALP in the range of healthy individuals could indicate that the animal is not likely to have EGUS at gastroscopy. However, this analyte is not able to differentiate between horses with EGUS and horses with other diseases, possibly because it can increase in inflammatory conditions and gastrointestinal diseases.

Aldolase activity was found to increase in those horses suffering from EGUS compared to healthy horses. Aldolase is involved in the impaired cell growth and proliferation of gastric epithelial cells, possibly due to the ability of aldolase to increase the hypoxia-inducible factor (HIF-1a) [29]. A previous study found that the presence of genes encoding forphospho-2-dehydro-3-deoxyheptonate aldolase protein was a risk factor for the development of peptic ulcer disease related to *Helicobacter pylori* in human beings [30]. The presence of aldolase in the saliva of horses with some type of EGUS could reflect the predisposition to altered gastric mucosa, although additional studies should be conducted in order to understand the behaviour of this protein in the ulceration process. The results of aldolase in our report contrast with a previous proteomic study [9] in which aldolase did not appear as a protein that changed between horses with ESSG or EGGD compared to healthy horses, and only changed between EGGD and ESGD. These differences could be explained by the assessment of protein abundance in the proteomic report in contrast to the enzymatic activity in the present study. Different behaviours between the amount of protein and its activity have been reported for other proteins, such as alpha amylase [31]. ROC analysis showed that salivary aldolase activity could be an aid to differentiate between healthy horses and animals with EGUS, although its sensitivity and specificity were lower than CALP.

A limitation of this manuscript is the lack of purified equine CALP as a standard in the assay, which would be preferable for this study. Currently, there is no international standard for CALP measurements [32]. Therefore, manufacturers currently rely on internally established standards, with different levels of standards among manufacturers depending on the chosen method, which will give variable values between assays. This is a common failing in assays for veterinary biomarkers [33]. It should be also pointed out that in case of a change in the batch of antibodies in the assay of CALP, a complete validation should be used in order to assure that the method works properly [34]. In addition, it would be interesting to elucidate the possible reasons for the increase in CALP and aldolase in saliva in this disease—whether it comes from serum or can be produced by the salivary glands. 

The analytes of this study, especially CALP, showed a potential to discriminate between horses with EGUS and healthy horses. However, it should be pointed out that these analytes were increased in horses with EGUS as well as in horses with other inflammatory processes, and this indicates that they cannot be used as discriminatory markers between EGUS and other diseases with similar clinical signs. Currently, the use of saliva biomarkers in EGUS is limited to research. Further prospective studies with a larger population of horses with ESGG and EGGD to evaluate the real practical use in the routine of saliva CALP and aldolase measurements, as well as their combined use with other biomarkers described in previous reports [6,24], should be performed to establish the value of a potential biomarker-guided assessment of EGUS and to explore the cost effectiveness of such an approach. 

## 5. Conclusions

Overall, CALP and aldolase can be measured in the saliva of horses using the assays validated in this study, which were demonstrated to be precise and accurate. In addition, CALP and aldolase showed changes in the saliva of horses with EGUS and could be considered as potential biomarkers of this disease, especially for differentiating horses with EGUS from healthy horses.

## Figures and Tables

**Table 1:** Precision of the calprotectin and aldolase assays in horse saliva.

Method	Comparison	Samples	Mean	SD	CV (%)
Calprotectin (mg/L)	Intra-assay	High	38.8	6.04	0.38
		Low	3.2	0.04	1.31
	Inter-assay	High	43.2	8.98	6.85
		Low	4.64	0.38	4.38
Aldolase (U/L)	Intra-assay	High	92.38	6.50	7.03
		Low	7.14	0.25	3.52
	Inter-assay	High	89.74	8.92	9.98
		Low	5.89	1.12	8.52

SD, standard deviation; CV, coefficient of variation.

**Table 2:** Recovery of calprotectin and aldolase measurements in horse saliva.

		Calprotectin Concentration			Aldolase Activity		
% Analyte		Expected (mg/L)	Observed (mg/L)	Recovery (%)	Expected (U/L)	Observed (U/L)	Recovery (%)
100	0	65	65	100	35	35	100
75	25	51.5	53.9	104.8	31.6	31.2	98.5
50	50	36.1	41.3	114.4	28.3	28	98.8
25	75	24.9	20.8	119.5	24.7	25	100.9
0	100	5.5	4.9	89.8	21.4	21.4	100

## Data Availability

The data presented in this study are available on request from the corresponding author for scientific purposes.

## Supplementary Materials

The following supporting information can be downloaded at: https://www.mdpi.com/article/10.3390/ani13081367/s1, Table S1: Final diagnoses of the 21 animals suspected of Equine Gastric Ulcer Disease with non-compatible gastroscopy.
