# Supplementary material for: Changes in Calprotectin (S100A8-A9) and Aldolase in the Saliva of Horses with Equine Gastric Ulcer Syndrome
Source: Animals (Basel). 2023 Apr 16;13(8):1367. doi: 10.3390/ani13081367 (PMC10135270; doi:10.3390/ani13081367)
Supplement: Supplementary file 1 [file animals-13-01367-s001.zip › animals-2267639-supplementary.pdf]

Table S1. Final diagnoses of the 21 animals suspected of Equine Gastric Ulcer disease with non-compatible gastroscopy.

Horse	Final diagnosis
1	Unspecific colic
2	Enteritis
3	Left dorsal displacement and impaction
4	Stress induced diarrhea
5	Gastric impaction
6	Dysbiosis
7	Flexure pelvic impaction
8	Sand and inflammatory bowel disease
9	Right dorsal displacement
10	Eosinophilic duodenitis
11	Head shaking and impaction
12	Upper airway viral infection
13	Chronic kidney failure
14	Fecal dysbiosis
15	Mild equine asthma
16	Unspecific colic
17	Chronic eosinophilic enteritis
18	Unspecific colic
19	Sand accumulation in colon
20	Epiploic foramen entrapment of small intestine
21	Unspecific colic
